# Development of a National Aboriginal and Torres Strait Islander Cancer Framework: A Shared Process to Guide Effective Policy and Practice

**DOI:** 10.3390/ijerph15050942

**Published:** 2018-05-09

**Authors:** Jenny Brands, Gail Garvey, Kate Anderson, Joan Cunningham, Jennifer Chynoweth, Isabella Wallington, Bronwyn Morris, Vikki Knott, Samantha Webster, Lauren Kinsella, John Condon, Helen Zorbas

**Affiliations:** 1 Menzies School of Health Research, Charles Darwin University, Darwin 0810, Australia; gail.garvey@menzies.edu.au (G.G.); kate.anderson@menzies.edu.au (K.A.); joan.cunningham@menzies.edu.au (J.C.); bronwyn.morris@menzies.edu.au (B.M.); burchett-knott@bigpond.com (V.K.); john.condon@menzies.edu.au (J.C.); 2 Cancer Australia, Surry Hills 2010, Australia; jennifer.chynoweth@canceraustralia.gov.au (J.C.); atsiprograms@canceraustralia.gov.au (I.W.); atsiprogram@canceraustralia.gov.au (S.W.); lauren.kinsella@canceraustralia.gov.au (L.K.); helen.zorbas@canceraustralia.gov.au (H.Z.)

**Keywords:** Indigenous health, cancer, framework, consultation, collaboration, research translation

## Abstract

Indigenous Australians experience a substantially higher cancer mortality rate than non-Indigenous Australians. While cancer outcomes are improving for non-Indigenous Australians, they are worsening for Indigenous Australians. Reducing this disparity requires evidence-based and culturally-appropriate guidance. The purpose of this paper is to describe an initiative by Cancer Australia and Menzies School of Health Research (Menzies) to develop Australia’s first National Aboriginal and Torres Strait Islander Cancer Framework using a process of co-design with relevant stakeholders. The initiative was guided by three core principles: achieving policy-relevant evidence-based outcomes; engaging and maintaining trust with Indigenous Australians at every phase; and employing best-practice and appropriate research methods. Four components of research comprised the Framework development: evidence review; multifaceted stakeholder consultation and input; triangulation of findings; and direct stakeholder input in drafting and refining the Framework. The evidence review confirmed the increasing burden of cancer on Indigenous Australians, while stakeholder consultations facilitated comprehensive input from those with lived experience. The consultations revealed issues not identified in existing literature, and gave different emphases of priority, thus reinforcing the value of including stakeholder perspectives. This paper focuses primarily on documenting the methods used; findings are presented only in order to illustrate the results of the process. The published Framework is available at www.canceraustralia.gov.au; further description and analyses of findings from the consultations will be published elsewhere. The logistics inherent in large-scale consultation are considerable. However, the quality of data and the foundation for sustained partnership with stakeholders and knowledge translation vastly outweighed the challenges. The process of wide-ranging stakeholder consultation described in this paper offers a model for other areas of national and international Indigenous priority setting and policy and practice development that meets the needs of those most affected. The Framework, through the establishment of an agreed, shared and evidence-based agenda, provides guidance for jurisdictional cancer plans, optimal care pathways, and program and service planning for the multiple players across all levels of the health system.

## 1. Introduction

Indigenous populations around the world experience poorer health compared to non-Indigenous populations [1]. Dispossession, marginalization and alienation from one’s culture have been identified as key factors underpinning the widespread health disparities between Indigenous and non-Indigenous people [1]. Other factors such as social disadvantage (e.g., lower levels of education and higher unemployment rates), higher smoking rates, poor nutrition, physical inactivity and poor access to health services contribute to Indigenous peoples’ health disadvantage.

Disparities are seen across many indicators of the health and wellbeing of Indigenous and non-Indigenous Australians [2], including a more than 10 year gap in life expectancy as at 2015 [3]. (The term ‘Indigenous Australians’ is used respectfully in this paper to refer to Australia’s Aboriginal and Torres Strait Islander peoples.) In 2008, Australia’s federal, state and territory governments joined non-government agencies and committed to a major initiative aimed at Closing the Gap in life expectancy and health status within a generation (by 2030) [4]. Subsequent policies and programs highlighted the need to address two overarching matters: the social and cultural determinants of health which are estimated to account for between one-third and a half of the gap in life expectancy [2,5,6], and the need to make health care accessible, culturally safe and appropriate, effective and responsive for all Indigenous Australians [7]. The Closing the Gap Agreement also emphasised the need for action that is based on evidence and ‘what works’ in Indigenous health.

Indigenous leaders have long argued that improvements to the processes of evidence and policy development, including increased attention to implementation, are critical to achieving improved outcomes in Indigenous health [8,9]. Repeated failure to have timely and constructive engagement with Indigenous stakeholders has been identified as a major barrier to change [10]. Internationally, there has been growing interest in scientific approaches to improve the implementation of evidence-based policy, programs and practice [11], in order to more systematically deliver better health and social outcomes. In Australia, the Indigenous health research sector has taken an active role in the emerging disciplines of knowledge translation and implementation science [11], and has identified key principles that make it more likely that research may translate into real benefits for Indigenous communities, families and individuals [12].

Cancer is a leading cause of death worldwide and a substantial and increasing burden of cancer is carried by Indigenous people, their families and communities. In Australia, the burden of cancer is high among Indigenous peoples [13]. In comparison to their non-Indigenous counterparts, Indigenous Australians experience higher age-standardised incidence and mortality rates for all cancers combined (484/439 per 100,000 and 221/171 per 100,000, respectively) [13]. The reasons for the cancer burden among Indigenous Australians are multifaceted. Factors such as a later presentation at diagnosis [14,15], a higher incidence of preventable, yet fatal cancers [13] being less likely to participate in cancer screening programs [16], and less likely to receive active treatment or be hospitalised for cancer [15,17] in comparison to non-Indigenous Australians have been reported as contributing to the poorer cancer outcomes.

In addition, due to the urban-centric nature of Australia’s health services and infrastructure, Indigenous Australians who live in regional and remote locations face added barriers to accessing optimal cancer prevention, detection and treatment services [18]. Cancer survival rates decrease for Indigenous Australians as their level of remoteness increases [14]. These unfavourable circumstances are cumulative and contribute to Indigenous Australians being less likely to survive five years after a cancer diagnosis than non-Indigenous Australians [17]. Of significant concern is the widening cancer mortality gap. Between 1998 and 2015, the cancer death rate for non-Indigenous Australians fell by 13%, while during the same period of time, it increased by 21% for Indigenous Australians [19].

In light of the burgeoning impact of cancer on Indigenous Australians, Australia’s national cancer control agency, Cancer Australia, initiated a process in 2014 to develop a national approach to address disparities and improve cancer outcomes for Indigenous Australians. Cancer Australia proposed the development of a National Aboriginal and Torres Strait Islander Cancer Framework (the Framework) as a mechanism to identify priorities and guide future cancer control efforts for Indigenous Australians.

The development of a national Framework would provide opportunities to consolidate the evidence base; identify priorities for action; and engage the diverse stakeholders whose input would be critical to achieving this goal. It was agreed that the Framework would be developed consultatively to incorporate published evidence, expert knowledge and the lived experience of Indigenous people, in order to deliver credible and culturally appropriate guidance to steer action and effect change across many levels of the health system and across Australia [10,20]. The involvement of a broad range of stakeholders throughout the process formed a significant component of Cancer Australia’s plan for developing the Framework. While stakeholder engagement is not uncommon in the co-design of community development projects, in health care co-design has largely been applied at the local level by involving consumers (patients and staff) in improving the service environment and patient experience, or developing consumer materials [21,22,23,24,25]. Co-design is less common in the development of macro level health policy, and in research it can be difficult to obtain funding or ethics approval for co-design projects [26]. Another barrier to the use of co-design methods is the considerable logistical challenge of engaging with geographically and culturally disparate and hard to reach stakeholders, although developments in interactive online data collection and social media make it increasingly possible to interact with large segments of the population in a short period of time [22,27].

This paper describes the development process of the Framework [28]. More than 350 individuals from key stakeholder groups across Australia’s diverse geographic, cultural and jurisdictional boundaries were directly involved. The engagement process provides a useful model for developing policy that responds to the needs and contexts of those most affected, and is informed by the practicalities of service delivery. This approach also optimises the engagement of stakeholders who can drive change in this area, thus fostering knowledge translation and improved policy and practice.

## 2. Methods

### 2.1. Project Management

Following a tender process, Menzies School of Health Research (Menzies) was contracted to develop the Framework in partnership with Cancer Australia. Menzies is one of Australia’s leading health research institutes, a not-for-profit organisation with a primary focus on improving the health and wellbeing of Indigenous Australians and extensive experience in developing sustained and meaningful partnerships with Indigenous Australian cancer survivors and others in cancer control.

The project was funded by Cancer Australia and managed as a partnership between Cancer Australia and Menzies [29]. Cancer Australia established a Project Steering Group (PSG) with 50% Indigenous membership, to provide guidance and advice on the process, content and structure of the Framework. Cancer Australia established three key principles to guide the development of the Framework: a collaborative approach between Cancer Australia and Menzies; a consultative approach with the broader cancer control and Indigenous communities; and an evidence-based approach to ensure that the Framework would be grounded in the best available evidence. Ongoing and respectful engagement with Indigenous Australians was embedded in every phase of developing the Framework; together with best-practice and appropriate research methods [12]. The Menzies project team used an evidence-based approach to achieving policy-relevant outcomes from research [30], guided by the following tenets:

- stakeholders (potential users of research) are involved throughout;
- research is outcomes-focused from the outset;
- syntheses of findings are used rather than one off reports;
- strong relationships exist between researchers and stakeholders;
- research targets multiple levels of change (i.e., does not simply operate in one domain);
- research is credible and of high quality.

### 2.2. Ethics Approval

The development of the Framework was carried out under a consultancy contract awarded following a Cancer Australia competitive tender process, and the consultations were required to meet the requirements of the Australian Privacy Act 1988 and the Australian Privacy Principles. Ethical approval for secondary analysis of data collected during the Framework consultations was granted by the Human Research Ethics Committee (HREC) of the Northern Territory Department of Health and Menzies School of Health Research (HREC Reference: 2016–2545), with the clear proviso that data would be deidentified and aggregated and not be used to identify individuals.

### 2.3. Framework Development

The four main research activities conducted to develop the Framework were: a review of existing evidence; stakeholder consultation and input; data synthesis and interpretation; and key stakeholder input and feedback on drafting the Framework.

#### 2.3.1. Evidence Review

A literature review of qualitative and quantitative evidence was conducted to identify the issues, gaps, priorities, strategies and evidence for improving cancer outcomes in Indigenous Australians. The results from the review informed the content of the stakeholder consultations and ultimately of the Framework. Population health data on Indigenous Australians, papers from peer-reviewed scientific journals and unpublished or ‘grey’ literature were included in this review.

The review was conducted in two phases. Menzies sub-contracted the International Centre for Allied Health Evidence (iCAHE) to carry out an initial robust literature review for Phase 1. iCAHE reviewed literature published from January 2010 to September 2014, building on a previously published review [31]. Phase 2 involved a second scan of the literature by the Menzies team, using the same search and exclusion criteria as iCAHE, but extending the publication date range to December 2014 to include a number of more recent items. The combined result of the two phases of the evidence review revealed 68 publications, including 17 from the grey literature.

A review of these items identified a large number of issues, barriers and enablers that affect cancer outcomes for Indigenous people, as well as 48 initiatives aimed at improving cancer outcomes for Indigenous Australians (including programs, production/distribution of resource material, and services).

#### 2.3.2. Stakeholder Consultations and Input

**Figure 1:**
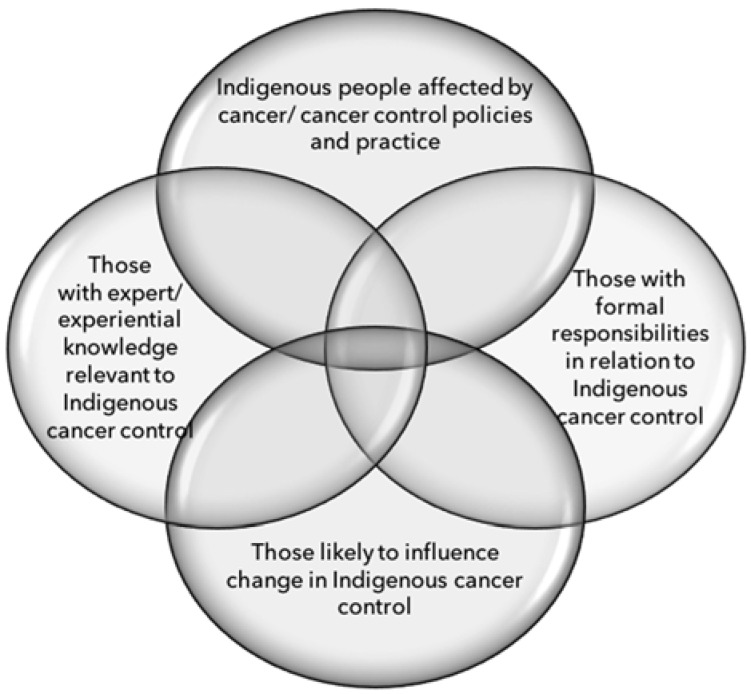
Categories of stakeholders identified to be necessary for robust policy development (adapted from [32]).

A detailed stakeholder mapping process was carried out to ensure that the consultations could achieve genuine engagement with the many communities, organisations and individuals whose efforts are required to improve cancer outcomes for Indigenous Australians. Specific stakeholder groups were identified across four categories (Figure 1). The interests and potential roles of each stakeholder group in the consultations (and in the eventual implementation of the Framework) were mapped (Table 1), drawing on the expert knowledge and networks of Menzies and Cancer Australia. Communication preferences were identified for each group (where evidence or experiential knowledge of preferences was available), along with suitable methods for achieving the desired levels of engagement.

Three consultation formats were selected, informed by the mapping process, the available budget and the project timeframe: regional forums, an online survey and online discussion boards. Engagement strategies were developed for each type of consultation, although an overarching approach was for the Menzies project team and Cancer Australia to leverage existing personal, professional or institutional relationships across the country to promote engagement with the appropriate target audiences.

In total, there were 465 episodes of participation (Table 2). Some participants took part in more than one form of consultation (i.e., in both a regional forum and the online survey). Almost half of all participants were Indigenous Australians, including many directly affected by cancer, their families and carers.

***Regional forums.*** Regional forums were seen as the most reliable consultation format for engaging the broadest range of Indigenous Australians, and a practical mechanism for gaining a snapshot of the way that cancer services are provided across diverse settings, services and infrastructure across Australia. Regional forums were held between February and March 2015 in six diverse geographical locations across Australian jurisdictions in New South Wales; Victoria; Northern Territory; Queensland; Western Australia; and the Torres Strait. An initial pool of potential forum sites were identified based on the availability of suitable target groups; accessibility during the tail end of the Wet Season across northern Australia; and the strength of existing relationships which could be leveraged to organise a forum within the project time constraints. Final forum sites were selected, and the events planned, organised and promoted in collaboration with an Indigenous health service/organisation in each location. Local promotions included consultations with men’s and women’s groups, distribution of electronic or printed flyers (supplied by the project) and radio interviews.

Each forum commenced with an Acknowledgement of the local Traditional Indigenous people and their land and/or a prayer ceremony from an Indigenous community Elder. The regional forums were facilitated by an experienced Indigenous facilitator, and the Menzies/Cancer Australia team provided specialist content input where needed.

Forum participants were asked to consider key issues identified through the evidence review: whether these issues tallied with their own experiences, if any key issues were missing, and which issues were most important. Participants were then invited to share their views on a proposed structure and use of a national Framework. In most locations, the regional forums were preceded by less-formal one-hour sessions that provided a ‘safe space’ for Indigenous Australians affected by cancer to talk privately with the Menzies/Cancer Australia team, in case they did not feel comfortable sharing their personal experiences at a more public gathering. Attendance at these sessions varied, from one or two attendees to six at one site.

In total, 121 participants attended the regional forums, of which 71 (59%) reported being of Aboriginal and/or Torres Strait Islander descent. A wide range of health services and health professionals attended the regional forums and participants were predominately female (78%).

***Survey.*** An online survey was developed based on the outcomes of the evidence review, regional forums and input provided by the PSG. The survey consisted of four sections:

1. Demographic information (e.g., age, sex, professional background, Indigeneity);
2. Issues, gaps and barriers for Indigenous Australians across the cancer continuum (including cancer prevention, screening and early detection, diagnosis and treatment, survivorship and living well after cancer, and palliative care);
3. System-wide factors, such as the quality and usability of data to support evidence-based action;
4. Development of the National Aboriginal and Torres Strait Islander Cancer Framework (its potential role/benefit/purpose).

Respondents were asked to rank the list of issues identified in earlier consultations in order of their perceived impact on cancer outcomes and identify any important issues missing from the list.

The survey was delivered via Survey Monkey (www.surveymonkey.com, San Mateo, California, USA) and was disseminated via email to a targeted group of stakeholders (individuals, professional bodies, associations, advocacy organisations, primary health care and cancer care services, Indigenous and non-Indigenous organisations). Cancer Australia’s and Menzies’ extensive networks and email lists were used to facilitate dissemination. A total of 852 invitations were sent via email with the link to the online survey.

A total of 326 people (38.3% response rate) completed the survey in the period from 25 February to 31 March 2015. Of these respondents, 36% identified as Indigenous Australians, 82% were female, and the majority were aged 45 to 64 years (55%) and resided in Queensland (29%), New South Wales (24%) or Victoria (14%). Participants could select multiple responses to appropriately describe their role or experience in cancer care, in health care provision and to which parts of the cancer continuum their experience related. As such, the percentages reported for professional roles signify the percentage of participants who selected that role. As they were not mutually exclusive and participants often work in more than one area of the cancer continuum, the percentages across roles is greater than 100%. Participants held a range of roles, with the largest reported role being health professionals (50%). The most common roles reported by health professionals were: working in cancer treatment (59%), followed by palliative care (41%). The remainder of reported roles were evenly represented across the cancer continuum.

***Online discussion.*** An online discussion platform was another mechanism used to engage stakeholder views about cancer control issues for Indigenous Australians. The National Indigenous Cancer Network’s (NICaN) ‘yarning place’ on the Australian Indigenous HealthInfoNet website (www.yarning.org.au/group/15) was used to host online discussions about issues and priorities for improving cancer outcomes for Indigenous Australians. NICaN is a partnership between Menzies, the Australian Indigenous HealthInfoNet, the Lowitja Institute and Cancer Council Australia and aims to ensure that evidence about cancer in Indigenous people is accessible to Indigenous audiences, consumers, service providers, researchers and health professionals from a broad range of disciplines, as well as private sector and government organisations.

Two methods were used to host online discussions. Five yarning topics (one for each stage of the cancer continuum) were established on the NICaN site. Menzies staff posted a broad question for each topic to trigger discussion and users were able to comment at any time during the period 11–25 March 2015. Secondly, Menzies facilitators/moderators and guest facilitators with lived or professional experience relevant to Indigenous cancer issues hosted three ‘live’ online discussions on the site. The response to the on-line engagement strategies was not strong. Out of a possible 299 yarning members at that time, only 6 people took part in the on-line discussion. The small number of participants may reflect the limited promotion of these sessions, improving online and social media engagement strategies for community consultation was a key learning area for the research team.

### 2.4. Data Synthesis and Interpretation

Quantitative and qualitative data gathered from the literature review and survey relating to issues, barriers and enablers to cancer control for Indigenous Australians were extracted and analysed and summarised at individual, organisational and health system levels. Qualitative data from the regional forums and online discussions were also thematically coded and summarised in relation to stages of the cancer continuum and at individual, organisational and health system levels.

Survey data was analysed using SPSS Statistics to weight ranking values and calculate average total scores for each issue in each section of the continuum. Items that individuals ranked ‘1’ (most important) were given a weighting of 15 (the highest number of issues listed in any of the ranking questions), rankings of ‘2’ were weighted as 14, and so on. This was a simple conceptual adjustment so that higher scores represented a higher ranking/priority. Issues were then ordered according to the highest average score.

The top issues affecting cancer outcomes for Indigenous people were analysed for the following respondent groups: all respondents; Indigenous respondents only; non-Indigenous respondents only; Indigenous consumers (patients, survivors, carers/family members, consumer advocates/ambassadors) only; and for subgroups of respondents relevant to each phase of the continuum (for example, health professionals who identified themselves as working in prevention). Responses to open-ended questions were thematically coded and analysed at individual, organisational and system levels.

All findings of the evidence review and the stakeholder consultations and input were reviewed and synthesised to highlight key issues that should be considered in developing the draft Framework and evidence-based priorities that would have the greatest impact on reducing disparities in cancer outcomes experienced by Indigenous Australians. The data from the various modes of consultation were analysed using the qualitative methods of thematic synthesis and triangulation. In this way, priorities across the different data collection methods were identified and defined, and then synthesised into a final priority list in the draft Framework.

### 2.5. Production of the Draft Framework

Following the evidence review and the stakeholder consultation, an iterative approach was used to prioritise the key issues based on the qualitative data from the consultations and develop a Discussion Paper and draft Framework. The approach was to identify issues, gaps and barriers, and then refine and prioritise these into principles, priorities and enablers; taking into consideration also contextual issues related to the Australian health system, Indigenous health and health care, and the social determinants of health. The PSG played an active role in the shaping of the principles and priorities of the draft Framework, drawing on the extensive experience and expert knowledge of its members. A theory of change model [33,34] was developed and included in the Discussion Paper. Priorities were considered in relation to relevant policy documents, including state and territory cancer plans, frameworks and strategies [35,36,37,38,39,40,41,42], profession-specific strategies [43,44], national strategies for cancer and/or Indigenous health [7,45,46,47,48], and key international policy papers [49,50,51]. A final list of seven key priority areas and enablers for action were included in the draft Framework.

Following the development of the draft Framework, a national forum was held in Sydney with 50 invited participants representing key stakeholders from governments, the Indigenous community controlled health sector, researchers, service providers and health professionals including Aboriginal and Torres Strait Islander Health Workers, and relevant peak bodies including cancer councils and consumer groups. The purpose of the national forum was to obtain validation and consensus on the priorities and enablers identified in the draft Framework, and to consolidate support in implementation of the final Framework. The draft Framework was circulated to invited participants in advance of the national forum with a survey to collect feedback.

Following this national forum, the Framework was finalised and launched by the then Australian Government Health Minister Sussan Ley in Broken Hill on the 31 August 2015 [28].

## 3. Results

While the aim of this paper is chiefly to describe the stakeholder consultation processes used in the development of the Framework, this section broadly describes the findings of the consultations that shaped the Framework. Specific details of the findings will be presented in future publications.

### 3.1. Evidence Review

The evidence review confirmed the increasing burden of cancer on Indigenous Australians, and also highlighted the specific cancers and cancer-related issues requiring the greatest attention. For example, factors that contribute to higher cancer mortality and poorer outcomes for Indigenous Australians include the high incidence of cancers that are largely preventable but also more likely to be fatal; lower rates of participation in cancer screening programs; and lower hospitalisation rates for cancer treatment than experienced by non-Indigenous Australians. The evidence review identified a large number of barriers and enablers that may affect Indigenous cancer outcomes. Forty-eight, mainly small-scale, initiatives aimed at improving Indigenous cancer outcomes were also identified; however, there was little high quality evidence of effectiveness or impact of these initiatives.

### 3.2. Stakeholder Consultations

In the main, the consultation results were consistent with the findings from the evidence review, particularly on the level of patient and community knowledge, fears and beliefs about cancer; a lack of cultural competence in cancer care support services and their workforce; and the impact of costs, transport issues, inappropriate accommodation and fears about experiencing cultural alienation on accessing or continuing treatment.

However, the consultations identified additional issues and revealed divergence in emphases and interpretations of topics or issues to those conveyed in the literature. Participants highlighted the need for more holistic care (for the person, not the disease); greater recognition of and support for carers and families; the importance of greater involvement of Indigenous people in planning, designing and evaluating cancer-related services at the local level; and the need for better coordination of care, particularly between sites or treatment phases. Participants in each form of consultation were asked to identify strategies or programs they considered to be positive and/or beneficial for Indigenous Australians. In comparison to the limited number of initiatives described in the scientific literature, participants gave many examples and descriptions of local organisations or practitioners that they perceived to be providing services that meet the needs of Indigenous people.

The preliminary regional forum sessions for those affected by cancer also provided opportunities to understand and document the day-to-day challenges many Indigenous Australians affected by cancer experience. Many of these challenges related to meeting the costs of undergoing cancer treatment, with some participants reporting they had sold or mortgaged their homes. Others spoke of the financial consequences of not working while undergoing treatment or recovery, an impact not only on salaries but on their retirement fund. In a similar vein, one participant in the larger regional forum spoke of her reluctance to seek help with cleaning or other domestic activities, for fear that such a request could end up involving welfare authorities and the risk of having her children taken into care.

### 3.3. Framework Development

The development of the Framework itself was iterative in nature and went through several co-design and consultation phases of review by the stakeholder groups. The resulting Framework identifies the priority areas that most require attention to better meet the needs of those most affected.

The following seven priority areas were identified via the consultation processes and included in the final Framework [28]:

1. Improve knowledge, attitudes and understanding of cancer by individuals, families, carers and community members (across the continuum);
2. Focus prevention activities to address specific barriers and enablers to minimize cancer risk for Indigenous Australians;
3. Increase access to and participation in cancer screening and immunisation for the prevention and early detection of cancers;
4. Ensure early diagnosis of symptomatic cancers;
5. Ensure Indigenous Australians affected by cancer receive optimal and culturally appropriate treatment, services, and supportive and palliative care;
6. Ensure families and carers of Indigenous Australians with cancer are involved, informed, supported and enabled throughout the cancer experience;
7. Strengthen the capacity of cancer related services and systems to deliver good quality, integrated services that meet the needs of Indigenous Australians.

## 4. Discussion

The development of the National Aboriginal and Torres Strait Islander Cancer Framework required a model of wide-ranging consultation with stakeholders rarely utilised in large research projects. The need to augment the available scientific research with insights from stakeholders’ lived experiences and expertise in dealing with Indigenous cancer care demanded a consultative approach that garnered many different perspectives. The development of the Framework involved a combination of research methods to maximize the range and depth of views from patients, their families, carers, health providers and government and community agencies. The consultative process employed in this project may guide other projects where solutions to similarly complex problems may be found through deep levels of stakeholder input, and ultimately ownership, and where significant changes to policy and practice are required.

The following discussion examines the efficacy, strengths and limitations of the process used in the development of this Framework.

### 4.1. Evidence Review Provides a Base for Consultation

The evidence review confirmed previous evidence of the increasing burden of cancer on Indigenous Australians, and also highlighted the specific cancers and cancer-related issues requiring the greatest attention. The review provided a strong starting point for the consultations with stakeholders, for example, providing structured discussion points for regional forums and the survey.

### 4.2. Benefits of Working through Established Networks and Respected Figures

A major strength of the project was the partnership between Menzies and Cancer Australia, two organisations with leadership and experience in nationally significant consultations and initiatives to improve cancer control in Australia. Cancer Australia’s leading role in the project, as a government organisation and leader in cancer control, gave the project credibility with stakeholders motivated by the opportunity to help bring about real change in policy and practice. The breadth and depth of Menzies’ and Cancer Australia’s existing partnerships and networks in cancer control facilitated consultations to ensure the project outputs were relevant, feasible and actionable. By choosing to work through local networks in organising the regional forums, the project team was able to ensure that local protocols were respected, and invitations reached the right audiences through trusted people or organisations, which further contributed to the creation of safe spaces to talk. The hands-on involvement of senior and well-respected Indigenous leaders in the consultations gave the project credibility among stakeholders that was critical in fostering effective and ongoing engagement. These are powerful benefits; the flip side of working through relationships so intensely is that great expectations are placed particularly on the Indigenous leaders involved.

### 4.3. Importance of Broad Stakeholder Consultation

The substantial time and effort involved in mapping the stakeholders and planning their engagement through established and trusted networks in preparation for the consultation process ensured the maximisation of diverse perspectives included in the analysis. This also created a milieu of inclusion, fundamental to fostering ownership and action for knowledge translation stemming from the Framework’s development. The benefit of this methodology was demonstrated by the overall consensus in ranking of priorities across the workshops. Although there was a high level of consistency in the ranking of priorities across the regional forums, the issues ranked highest differed between settings. For example, in major cities attendees were more likely to rank issues around costs as the number one priority while people in regional/remote areas were more likely to identify lack of support services and cultural competence in the health system as their top priorities. This reinforced the importance of consulting widely across geographical locations and using a range of consultative modalities.

### 4.4. Stakeholder Consultations Extend the Evidence-Base and Allow Drilling-Down

The consultations with stakeholders provided both validation and expansion of the findings from the evidence review. The smaller, private sessions with people affected by cancer provided an opportunity to learn about more personal aspects of the cancer experience than might be discussed in a public regional forum about priorities, and provided a safe space to hear the voices of those who may not otherwise been willing to speak up. These sessions were resource intensive, particularly if more than two or three people attended. However, the benefit of including the views and experiences of Indigenous people affected by cancer into the development of the Framework ensures that the needs, values and preferences of this population will guide future research, policy and practice.

### 4.5. Triangulation Using Multiple Research Methods and Perspectives

The consultation process for the Framework development purposely invited stakeholders to be involved in multiple research methods of consultation, including participation in both the survey and the public regional forums. This enabled multiple engagements with the same stakeholders using different methods to maximise the opportunities for participants to fully communicate their views and experiences. The variety of research methods used was successful in ensuring that data could be iteratively built upon to guide each successive wave of consultation. Additionally, the inclusion of a variety of stakeholder groups meant that the consultation included different perspectives on the same situations and problems. These strategies enabled the triangulation of data, which is conventionally believed to increase the validity of qualitative research and provide richer data to inform the research outcomes [52], and enabled the team to collect a more complete picture of cancer care for Indigenous Australians.

### 4.6. Building a Conversation, Building Momentum

The various methods used and commitment to engaging all relevant stakeholders throughout the development process facilitated consensus on priorities, and a high level of willingness to support its implementation. This was evident in the final consultation/feedback workshop, where nearly 50 leaders from key stakeholder organisations enthusiastically welcomed the development of the Framework to guide their work, and described specific actions that they would take to implement it. The resulting Framework can inform future policy and practice across a range of sectors, with the potential to bring about more person-centred, equitable, timely, effective and affordable care and services, and ultimately to improved outcomes for Indigenous Australians with cancer.

A Framework is open enough and flexible enough for different jurisdictions to respond as appropriate, avoiding a one-size-fits-all approach. The development of a Framework, rather than a set of specific goals or targets, provided opportunity for stakeholders at all levels to identify their interests in the Framework, and/or find guidance about priorities and plans for action.

While the benefits of this consultative approach are manifold, there are some limitations that must be acknowledged. This style of research can rarely engage with all stakeholders and those individuals who are in remote settings, are non-English speaking or less health-literate are less likely to have voluntarily engaged in this research process. Therefore, it is possible that some problems may have been underestimated or missed. The networks used to communicate to participants may not include all stakeholders and may skew the perspectives and priorities included in the Framework. However, the use of multiple different consultation modes (face to face and on-line) was intended to help ameliorate this limitation, and where possible, data was analysed for any differences among sub-groups: male, female Indigenous survey respondents for example.

Another limitation to this consultative approach may be the resources required. These consultations occurred over just a few months, and much of the budget went on travel costs for regional forums. The databases of existing contacts of Cancer Australia and Menzies are resources that have been built up over many years, and require regular updating to maintain currency. Similarly the credibility and influence of Indigenous leaders, and the relationships between them, are resources built up over time, almost impossible to replicate, but crucial to achieving the outcomes of these consultations.

## 5. Conclusions

While this paper describes the development of a Framework to address the specific cancer-related needs and priorities of Indigenous people in Australia, the planning and processes described may have wider application within Australia and internationally. There are substantial logistical challenges in working this way, but the gathering of the views, knowledge and lived experience of such a broad range of stakeholders, for the express purpose of developing national policy, and the rich quality of the ensuing data, laid a solid foundation on which to build ongoing partnership and engagement. The highly consultative approach enabled the development of the National Aboriginal and Torres Strait Islander Cancer Framework to be grounded in the experiences and knowledge of stakeholders that make it well-placed to guide effective change to improve outcomes for Indigenous Australians with cancer, now and into the future.

## Figures and Tables

**Table 1:** Stakeholder contributions and functions in consultation process.

Stakeholder Category	Stakeholder Groups	Contribution/Role
Indigenous people affected by cancer/cancer control	Cancer survivors, their families, communities, carers, advocates, support groups	Right to be involved in development of policies and programs that affect them Including the lived experience of people affected by cancer—vitally important to ensuring development of a robust and effective Framework Consumer view of where change needs to occur Personal stories that help others understand the patient experience and may help drive action Understanding of community-level conditions and what might be feasible or not
Those with formal responsibilities in relation to Indigenous cancer control	Cancer Australia, Australian Department of Health, State/territory health departments, Cancer councils, Cancer centres, Screening services, Australian Institute of Health and Welfare Regulators, legislators	Understanding the formal responsibilities and roles in the cancer control landscape Capacity to block or facilitate change Capacity to garner resources Important that these stakeholders recognise their responsibilities and are inspired to support change
Those with expert/experiential knowledge relevant to Indigenous cancer control	Health care professionals providing care to Indigenous people affected by cancer, Those involved in providing supportive services to Indigenous people affected by cancer Those who are involved in promoting healthy lifestyles (including tobacco control programs, healthy eating and exercise), Researchers , Indigenous primary health care providers, People with expertise in bringing about change in health systems, improvements in practice and behaviour change, Palliative care providers	Important to ensuring development of a robust and effective Framework Likely to be involved in implementing the Framework
Those likely to influence change in Indigenous cancer control	Champions of any sort, Health care professionals who are passionate about improving cancer outcomes for Indigenous people, Government health departments, Cancer Australia, Cancer Councils, Cancer advocates, Professional bodies of health professionals who interact with Indigenous people across the cancer continuum, Researchers and research funders	Buy in for Framework and its implementation Important that these stakeholders recognise the role they might play and are inspired to support change

**Table 2:** Numbers of participants in stakeholder consultations.

Consultation Format	Total No. of Participants #	No. and % Indigenous People	No. and % Indigenous Patients, Family Members and Carers
Six regional forums	121	71 (59%)	41 (34%)
National survey	326	118 (36%)	43 (13%)
Online discussion boards	18	n/a **	n/a **

# Participants may have contributed to more than one consultation format. ** This information was not collected.

## Supplementary Materials

Supplementary File 1

## References

[1] United Nations State of the World’s Indigenous Peoples. New York: United Nations Department of Economic and Social Affairs, Division for Social Policy and Development, Secretariat of the Permanent Forum on Indigenous Issues, 2009. http://www.un.org/esa/socdev/unpfii/documents/SOWIP/en/SOWIP_web.pdf.

[2] Commonwealth of Australia and Department of the Prime Minister and Cabinet (2017). Closing the Gap Prime Minister’s Report.

[3] Australian Institute of Health and Welfare (2015). AIHW: Trends in Life Expectancy. http://www.aihw.gov.au/deaths/life-expectancy/.

[4] Australian Human Rights Commission (2008). Close the Gap: Indigenous Health Equality Summit-Statement of Intent. https://www.humanrights.gov.au/publications/close-gap-indigenous-health-equality-summit-statement-intent.

[5] Booth A., Carroll N. (2005). The Health Status of Indigenous and Non-Indigenous Australians.

[6] Australian Institute of Health and Welfare (2014). Australia’s Health 2014.

[7] Commonwealth of Australia (2013). National Aboriginal and Torres Strait Islander Health Plan 2013–2023.

[8] Otim M.E., Kelaher M., Anderson I.P., Doran C.M. (2014). Priority setting in Indigenous health: Assessing priority setting process and criteria that should guide the health system to improve Indigenous Australian health. Int. J. Equity Health.

[9] Humphery K. (2001). Dirty questions: Indigenous health and ‘Western research’. Aust. N. Z. J. Public Health.

[10] Hunt J. (2013). Engaging with Indigenous Australia—Exploring the Conditions for Effective Relationships with Aboriginal and Torres Strait Islander Communities.

[11] Wensing M., Oxman A., Baker R., Godycki-Cwirko M., Flottorp S., Szecsenyi J., Grimshaw J., Eccles M. (2011). Tailored Implementation for Chronic Diseases (TICD): A project protocol. Implement. Sci..

[12] Jamieson L.M., Paradies Y.C., Eades S., Chong A., Maple-Brown L., Morris P., Bailie R., Cass A., Roberts-Thomson K., Brown A. (2012). Ten principles relevant to health research among Indigenous Australian populations. Med. J. Aust..

[13] Australian Institute of Health and Welfare (2017). Burden of cancer in Australia: Australian Burden of Disease Study 2011.

[14] Diaz A., Whop L.J., Valery P.C., Moore S.P., Cunningham J., Garvey G., Condon J.R. (2015). Cancer outcomes for Aboriginal and Torres Strait Islander Australians in rural and remote areas. Aust. J. Rural Health.

[15] Moore S.P., Green A.C., Bray F., Garvey G., Coory M., Martin J., Valery P.C. (2014). Survival disparities in Australia: An analysis of patterns of care and comorbidities among indigenous and non-indigenous cancer patients. BMC Cancer.

[16] Roder D., Webster F., Zorbas H., Sinclair S. (2012). Breast screening and breast cancer survival in Aboriginal and Torres Strait Islander women of Australia. Asian Pac. J. Cancer Prev..

[17] Australian Institute of Health and Welfare and Cancer Australia (2013). Cancer in Aboriginal and Torres Strait Islander Peoples of Australia: An Overview.

[18] Angus S. (2006). Queensland Aboriginal and Torres Strait Islander Women's Cervical Screening Strategy 2006–2010.

[19] Australian Institute of Health and Welfare and AACR (2017). Cancer in Australia: An Overview 2017, in Cancer.

[20] Lencucha R., Kothari A., Hamel N. (2010). Extending Collaborations for Knowledge Translation: Lessons from the Community-Based Participatory Research Literature.

[21] Robert G., Cornwell J., Locock L., Purushotham A., Sturmey G., Gager M. (2015). Patients and staff as codesigners of healthcare services. BMJ.

[22] Boyd H., McKernon S., Mullin B., Old A. (2012). Improving health care through the use of co-design. N. Z Med. J..

[23] Nilsen E.S., Myrhaug H.T., Johansen M., Oliver S., Oxman A.D. (2006). Methods of consumer involvement in developing healthcare policy and research, clinical practice guidelines and patient information material. Cochrane Database Syst. Rev..

[24] Castro E.M., Malfait S., Van Regenmortel T., Van Hecke A., Sermeus W., Vanhaecht K. (2018). Co-design for implementing patient participation in hospital services: A discussion paper. Patient Educ. Couns..

[25] International Collaboration for Participatory Health Research (ICPHR) (2013). Position Paper 1: What Is Participatory Health Research?.

[26] Goodyear-Smith F., Jackson C., Greenhalgh T. (2015). Co-design and implementation research: Challenges and solutions for ethics committees. BMC Med. Ethics.

[27] Stelzle B., Jannack A., Noennig J.R. (2017). Co-design and co-decision: Decision making on collaborative design platforms. Procedia Comput. Sci..

[28] Cancer Australia (2015). National Aboriginal and Torres Strait Islander Cancer Framework.

[29] Zorbas H., Elston J. (2016). Sharing the challenge of cancer control for Indigenous Australians: A national agenda. Eur. J. Cancer Care (Engl.).

[30] Brands J., Gooda M. (2006). Putting the users of research in the driver’s seat: The Cooperative Research Centre for Aboriginal Health's new approach to research development. Aust. Aborig. Stud..

[31] Miller J., Knott V., Wilson C., Cunningham J., Condon J., Roder D., Cancer Service Networks National Demonstration Program, Cancer Council South Australia (2010). Aboriginal and Torres Strait Islander Cancer Control Research Project.

[32] Krick T., Forstater M., Monaghan P., Sillanpaa M. (2005). From Words to Action: The Stakeholder Engagement Manual. Volume 2: The Practitioner’s Handbook on Stakeholder Engagement. http://www.unep.fr/shared/publications/pdf/webx0115xpa-sehandbooken.pdf.

[33] Weiss C., Connell J.P., Kubisch A.C., Schorr L.B., Weiss C.H. (1995). Nothing as practical as good theory: Exploring theory-based evaluation for comprehensive community initiatives for children and families. New Approaches to Evaluating Community Initiatives: Concepts, Methods, and Contexts.

[34] Andersen A. (2004). A Community Builder’s Approach to Theory of Change: A Practical Guide to Theory Development.

[35] State Government Department of Human Services (2009). Providing Optimal Cancer Care: Supportive Care Policy for Victoria.

[36] Northern Territory Department of Health (2013). Northern Territory Cancer Plan 2013–2016.

[37] Department of Health and Human Services (2010). Tasmanian Cancer Framework and Strategic Cancer Plan 2010–2013.

[38] Queensland Health (2014). Cancer Care Statewide Health Service Strategy.

[39] Department of Health (2011). Western Australia Cancer Plan 2012–2017.

[40] Cancer Institute NSW (2010). NSW Cancer Plan 2011–15.

[41] Queensland Health (2009). Strategic Directions for Cancer Prevention and Control 2009–2012.

[42] Cancer Council SA and SA Health (2011). Statewide Cancer Control Plan 2011–2015.

[43] Royal Australian and New Zealand College of Radiologists (2012). Planning for the Best: Tripartite National Strategic Plan for Radiation Oncology 2012–2022 Version 1 2012.

[44] Palliative Care Australia (2011). Improving Access to Quality Care at the End of Life for Aboriginal and Torres Strait Islander Australians: Position Statement.

[45] National Health Priority Action Council (NHPAC) (2006). National Service Improvement Framework for Cancer.

[46] Health Workforce Australia (2013). National Cancer Workforce Strategic Framework.

[47] Cancer Australia and Cancer Voices Australia (2011). National Framework for Consumer Involvement in Cancer Control.

[48] Australian Health Ministers Advisory Council (AHMAC) (2004). Cultural Respect Framework for Aboriginal and Torres Strait Islander Health, 2004–2009.

[49] OECD (2013). Cancer Care: Assuring Quality to Improve Survival.

[50] World Health Organization (2002). National Cancer Control Programmes: Policies and Managerial Guidelines.

[51] Institute of Medicine (2013). Delivering High-Quality Cancer Care: Charting a New Course for a System in Crisis.

[52] Patton M.Q. (1999). Enhancing the quality and credibility of qualitative analysis. Health Serv. Res..

