# Supplementary material for: Development of a National Aboriginal and Torres Strait Islander Cancer Framework: A Shared Process to Guide Effective Policy and Practice
Source: Int J Environ Res Public Health. 2018 May 9;15(5):942. doi: 10.3390/ijerph15050942 (PMC5981981; doi:10.3390/ijerph15050942)

Supplementary Materials

Table 1: Stakeholder contributions and functions in consultation process

Stakeholder category	Stakeholder groups	Contribution /role
Indigenous people affected by cancer/ cancer control	Cancer survivors, their families, communities, carers, advocates, support groups	• Right to be involved in development of policies and programs that affect them<br>• Including the lived experience of people affected by cancer - vitally important to ensuring development of a robust and effective Framework<br>• Consumer view of where change needs to occur<br>• Personal stories that help others understand the patient experience and may help drive action<br>• Understanding of community-level conditions and what might be feasible or not
Those with formal responsibilities in relation to Indigenous cancer control	Cancer Australia, Australian Department of Health, State/territory health departments, Cancer councils, Cancer centres, Screening services, Australian Institute of Health and Welfare Regulators, legislators	• Understanding the formal responsibilities and roles in the cancer control landscape<br>• Capacity to block or facilitate change<br>• Capacity to garner resources<br>• Important that these stakeholders recognise their responsibilities and are inspired to support change
Those with expert/ experiential knowledge relevant to Indigenous cancer control	Health care professionals providing care to Indigenous people affected by cancer, Those involved in providing supportive services to Indigenous people affected by cancer<br>Those who are involved in promoting healthy lifestyles (including tobacco control programs, healthy eating and exercise), Researchers , Indigenous primary health care providers, People with expertise in bringing about change in health systems, improvements in practice and behaviour change, Palliative care providers	• Important to ensuring development of a robust and effective Framework<br>• Likely to be involved in implementing the Framework
Those likely to influence	Champions of any sort, Health care	• Buy in for Framework and its implementation

Stakeholder category	Stakeholder groups	Contribution /role
change in Indigenous cancer control	professionals who are passionate about improving cancer outcomes for Indigenous people, Government health departments, Cancer Australia, Cancer Councils, Cancer advocates, Professional bodies of health professionals who interact with Indigenous people across the cancer continuum, Researchers and research funders	• Important that these stakeholders recognise the role they might play and are inspired to support change

Table 2: Numbers of participants in stakeholder consultations

Consultation format	Total no. of participants#	No. and % Indigenous people	No. and % Indigenous patients, family members and carers
Six regional forums	121	71 (59%)	41 (34%)
National survey	326	118 (36%)	43 (13%)
Online discussion boards	18	n/a**	n/a**

Participants may have contributed to more than one consultation format
** This information was not collected

Figure 1: Categories of stakeholders identified to be necessary for robust policy development (adapted from Krick, H. et al. 2005. From Words to Action: The Stakeholder Engagement Manual, AccountAbility, the United Nations Environment Programme, and Stakeholder Research Associates, accessed online at www.unep.fr)

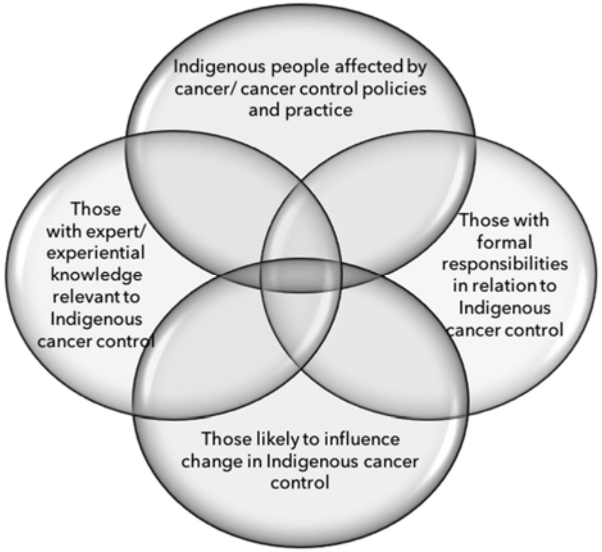

Supplement: Supplementary File 1 [file ijerph-15-00942-s001.pdf]
